# Chinese Medicine Jiedu Huayu Granules Reduce Liver Injury in Rats by Regulating T-Cell Immunity

**DOI:** 10.1155/2019/1873541

**Published:** 2019-11-27

**Authors:** Minggang Wang, Dewen Mao, Hanmin Li

**Affiliations:** ^1^Hubei University of Chinese Medicine, Wuhan, Hubei, China; ^2^The First Affiliated Hospital of Guangxi University of Chinese Medicine, Guangxi, China; ^3^Affiliated Hospital of Hubei University of Chinese Medicine, Wuhan, Hubei, China

## Abstract

Liver injury, one of the causes of liver failure, is mainly due to T-cell-mediated immunity. Traditional Chinese medicine Jiedu Huayu granules are often used to suppress liver damage and improve liver function. The specific regulatory mechanism of Jiedu Huayu granules has not been fully studied, and its function in the immune system remains unclear. Therefore, in this study, the mechanism of Jiedu Huayu granules in the prevention of hepatic injury was studied in a rat model of hepatic injury induced by D-galactoside and lipopolysaccharide. The cytotoxic T lymphocytes (CTLs) in the peripheral blood were examined. Perforin, granule B, and PD1 expression in CTL increased after the induction of hepatic injury and could be reduced by Jiedu Huayu granules. Hepatic apoptotic factors OX62, FAS, and TNFR1 associated with CTL function were also reduced by Jiedu Huayu granules. These results suggested that Jiedu Huayu granules could inhibit the inflammatory response to relieve liver damage by mediating the T-cell immunity. Therefore, the discovery of the mechanism of action of Jiedu Huayu granules in the immune system could allow their use more effectively in clinical practice.

## 1. Introduction

The liver is an important organ with metabolic functions in the human body. In the process of combating foreign viruses and bacteria, the liver acts as the first line of defense through an inflammatory reaction to resist the antigen invasion. However, an excessive inflammatory reaction often leads to hepatocyte apoptosis, liver damage, liver disease, viruses, alcohol, and formation of lipid peroxidation products, and various drugs may cause hepatitis [1]. Severe hepatitis can even cause acute liver failure (ALF), and although liver transplantation can alleviate and improve the condition, ALF mortality can be as high as 50% [2]. Viral hepatitis is an infectious disease caused by various hepatitis viruses. It can develop as acute hepatitis or chronic hepatitis. The patients may have clinical symptoms such as fever with jaundice, hepatomegaly, and liver damage. The patients with chronic hepatitis may develop liver fibrosis and end-stage cirrhosis with complications such as portal hypertension, liver failure, and increased incidence of liver cancer [3].

The D-galactoside and lipopolysaccharide-induced liver injury model induced in rats by toxic doses mimic viral hepatitis and is therefore widely used in drug screening studies against viral hepatitis and fulminant hepatic failure [4–6]. In this model, LPS stimulates Kupffer cells (specialized macrophages located in the liver) to secrete proinflammatory factors such as tumor necrosis factor TNF-*α* and various interleukins, and a series of inflammatory responses are the main causes of liver damage [7–9]. T-cell-mediated immune response is the main cause of liver injury. CD8^+^ cytotoxic T lymphocytes (CTLs) can cause acute necrotizing liver disease, and its killing effect on cells is mainly achieved by releasing perforin and granule B [10]. In chronic viral hepatitis, a high expression of the immunosuppressive molecule PD-1 and an autoimmune function on cells can also be detected [11, 12].

Jiedu Huayu granule is a traditional Chinese medicine consisting of 6 herbs, such as fungus Yin Chen, Hedyotis diffusa, red peony, rhubarb, turmeric, and scutellaria. A previous report demonstrated its successful use as a treatment against ALF as early as 20 years ago [13]. Previous studies showed that this drug can inhibit hepatocyte apoptosis by reducing intracellular protease expression, and it also has a palliative effect on hepatic encephalopathy complicated by liver injury [14]. Despite the evidence already reported on the effect of Jiedu Huayu granules, their specific regulatory mechanism on improving liver function has not been fully studied and remains still unclear [15]. Thus, our work focused on the evaluation of the effect of Jiedu Huayu granules on liver function, and our results suggested that it inhibited the inflammatory response to relieve liver damage by mediating the T-cell immunity.

## 2. Materials and Methods

### 2.1. Animals

Male rats (*n* = 136, 250 ± 30 grams) were purchased from the Hubei Experimental Animal Center (Hubei, China) and housed in a standard animal laboratory with a 12 h-12 h light-dark cycle. They were provided with water and standard rat chow ad libitum and randomly divided into three groups: control (*n* = 41), GalN/LPS-induced ALF (*n* = 55), and treatment (*n* = 40).

### 2.2. Establishment of ALF Model and Collection of Liver Tissue

The ALF model was established as previously described [15], with slight modifications. Three days before GalN/LPS model establishment, rats in the treatment group were treated with Jiedu Huayu granules by gavage at a concentration of 8.8 g/kg once every 12 hours for 6 days. The model group was treated with an equal volume of saline instead of traditional Chinese medicine according to the same protocol above. Approximately 24 h after GalN/LPS, rats were sacrificed and the liver was immediately perfused through the left ventricle with chilled saline containing 25 U/mL heparin. The liver tissue was harvested in tubes and immediately snap-frozen in liquid nitrogen. After 2 h, the liver samples were transferred to −80°C and stored at until analysis. Serum biochemistry and liver histopathology were used to assess liver injury.

### 2.3. Chinese Herbal Formula

Jiedu Huayu granules contain Yin Chen 30 g, Hedyotis diffusa 30 g, Radix Paeoniae rubra 50 g, rhubarb 15 g, Tulip 15 g, and Acorus tatarinowii 15 g. Traditional Chinese herb granules manufactured by Jiangyin Tian Jiang Pharmaceutical Co. Ltd. (Jiangsu, China) were purchased from the First Affiliated Hospital of Guangxi University of traditional Chinese Medicine. Quality control of Jiedu Huayu granule prescriptions was performed in accordance with a published article [15].

### 2.4. Flow Cytometry

Cells were washed in PBS twice and stained for cell surface CD3 or CD8 on ice for 20 min in PBS plus anti-CD3-FITC and anti-CD8-APC (BD). Stained cells were sorted using a BD FACSAria and analyzed using a BD LSRFortessa. FlowJo software (FlowJo LLC, Ashland, OR) was used to perform the flow cytometry analysis.

### 2.5. Western Blotting

Western blot analysis was performed as described. Cells were lysed on ice for 30 min. Approximately 50–150 mg of thermally denatured protein extract was loaded on a 10% polyacrylamide gel, electroblotted onto a nitrocellulose membrane, and blocked for one hour. The membrane was then incubated with antibodies against perforin, granule B, and PD1. Bands were visualized using the ECL Western blotting system (Santa Cruz Biotechnology).

### 2.6. Immunohistochemistry

Selected liver sections were deparaffinized, rehydrated, and heated in a microwave oven in 0.01 M citrate buffer (pH 6.0; Química Contemporânea, Diadema, Brazil) for 30 min. Endogenous peroxidase activity was blocked by 3% hydrogen peroxide for 10 min, followed by a wash with PBS. Sections were incubated overnight at 4°C with the following primary antibodies: anti-FAS (1 : 50), anti-OX62 (1 : 10), and anti-TNFR1 (1 : 100). The primary antibody was then detected using avidin-biotin peroxidase detection solution (DakoCytomation labelled streptavidin biotin reagent; DakoCytomation, Glostrop, Denmark; and System-horseradishperoxidase; Dako, Glostrop, Denmark), and the signal was visualized using diaminobenzidine (DakoCytomation) and Substrate Chromogen System (Dako). Slides were counterstained with Harris's hematoxylin, dehydrated, cleared, and mounted. Positive controls from the appendix and tonsils were used. Cells were initially observed at a low magnification (×100) to assess the general distribution of the primary antibody. Samples were subsequently examined at a higher magnification (×400) [16]. Liver cells (exhibiting gross and evident nucleoli and irregular chromatin) were identified, and stained cells were counted at the higher magnification.

### 2.7. Real-Time Quantitative PCR (RT-qPCR)

cDNA templates were prepared after RNA extraction and reverse transcription. Amplification was performed on a real-time PCR system (Applied Biosystems 7500, USA). The whole procedure was according to the manual of SYBR® Premix Ex Taq™ kit (Takara RR420A, Japan). Relative expression was calculated using formula 1/2△△Ct. Primers used for detecting the different expression of specific genes were as follows: GAPDH: forward 5′-ACAGCAACAGGGTG‐GTGGAC-3′, reverse 5′-TTTGAGGGTGCAGCGAACTT-3′. perforin: forward 5′-GCTGGATGTGAACCCTAAACC-3, reverse 5′-GGAGCTGTTAAAGTTGTGGGG -3′. GrB: forward 5′-AGTGTGGCGGCTTCCTTATA-3′, reverse 5′-TATACGCTGGGTGGGGAATG-3′. PD1: forward 5′-GG‐TATGTCAGAGGCCAGAGAA-3′, reverse 5′-AATGG‐TGGCGTATTCTGTGTG -3′.

### 2.8. Statistical Methods

Data were subjected to statistical analysis using the statistical package for social sciences (SPSS), version 18.00. A one-way ANOVA or Student's *t*-test was used to determine statistical significance. A probability of *P* < 0.05 was considered statistically significant. All data are presented as mean ± SD.

## 3. Results

### 3.1. Jiedu Huayu Granules Chinese Medicine Reduced the Proportion of CTL Cells

**Figure 1:**
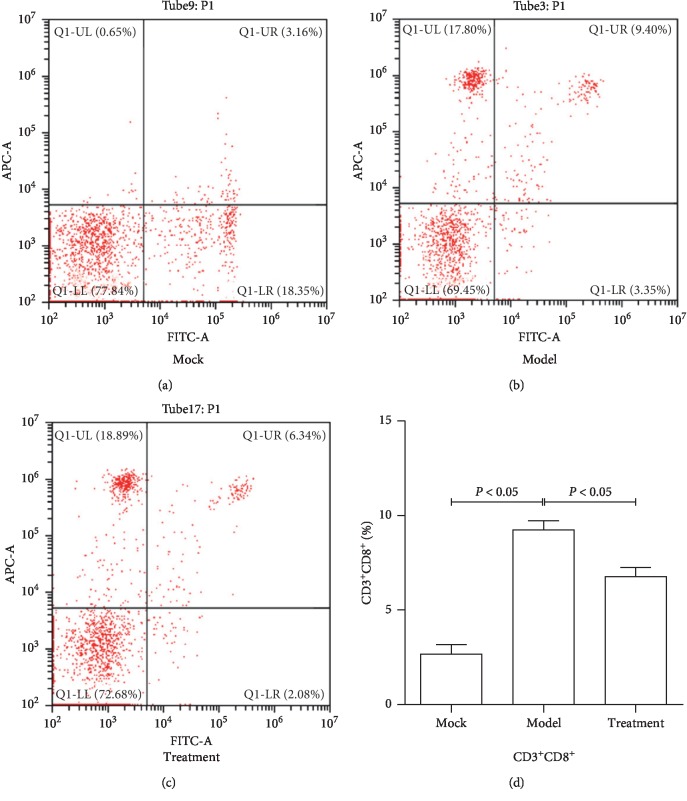
Effect of Jiedu Huayu granules on rat CTL cells. (a–c) CD3^+^CD8^+^ cells from control group, model group, and treatment group by flow cytometry. The proportion of CD3^+^CD8^+^ cells was detected at the same time. (d) Bar graph showing CD3^+^CD8^+^ cell percentage in the three groups.

In patients with ALF, CTL mediated by immune function can lead to an overimmune response, leading to liver failure. Therefore, in order to detect the changes in CTL cells in ALF and Jiedu Huayu granule treatment, six rats were randomly selected from each group to detect the proportion of CTL cells in peripheral blood cells by flow cytometry. The results showed that, in the control group, the percentage of CD3^+^CD8^+^ CTL cells in rat peripheral blood was 3.16%, while in the ALF model group, the percentage of CD3^+^CD8^+^ CTL cells increased to 9.40%. After treatment with Jiedu Huayu granules, the percentage of CD3^+^CD8^+^ CTL cells decreased significantly to 6.34% (Figure 1). These experimental results have been described in our previous article [17] and indicate that Jiedu Huayu granules can reduce the proportion of CTL cells in the process of alleviating ALF, suggesting that Jiedu Huayu granules can alleviate excessive immune stress by reducing the ratio of CTL cells in the pathological process of acute liver injury.

### 3.2. Jiedu Huayu Granule Chinese Medicine Inhibited CTL Function

**Figure 2:**
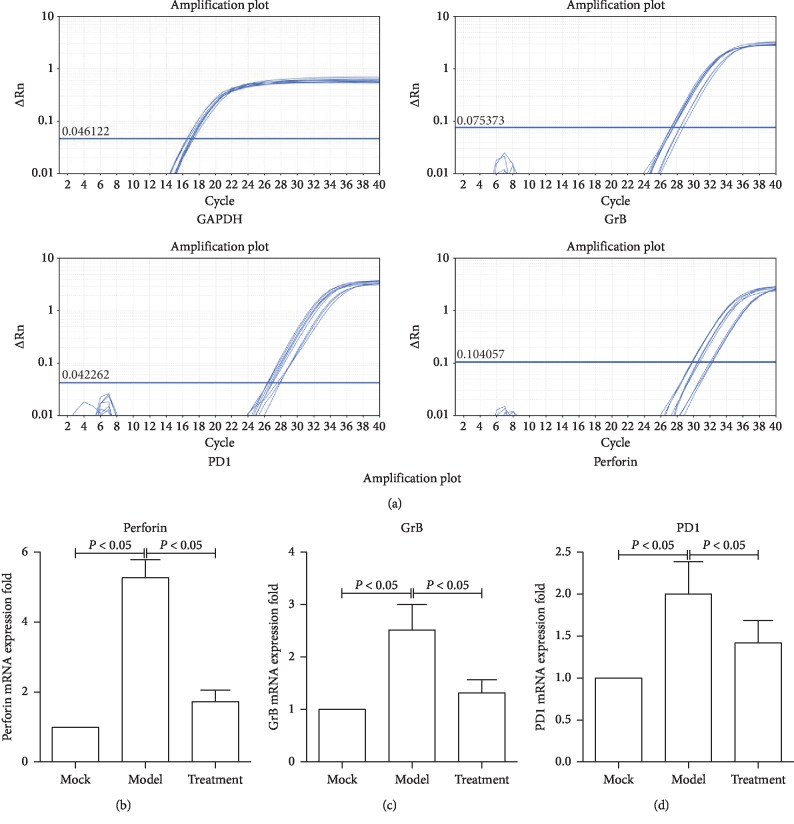
The expression of genes related to cell killing was detected by RT-qPCR. CD3^+^CD8^+^ cells were isolated, and total RNA was extracted. The expression of perforin, GrB, and PD1 mRNA was detected by RT-qPCR after reverse transcription. Each experiment was repeated three times.

**Figure 3:**
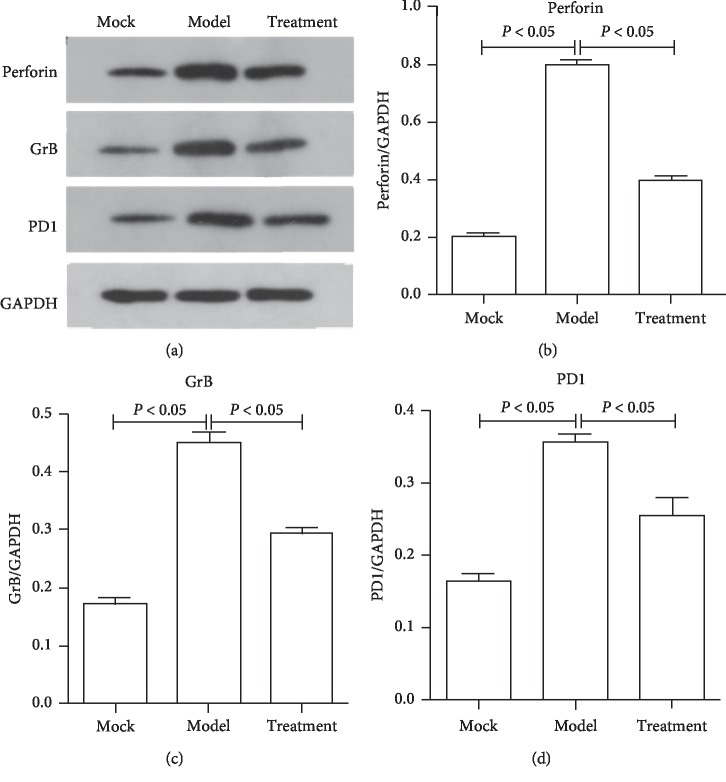
The expression of proteins related to cell killing was detected by using western blot. CD3^+^CD8^+^ cells were isolated and total proteins were extracted. Perforin, GrB, and PD1 protein expression was detected by using western blot. Quantification of the gray bands was performed using BandScan software.

Ten rats from the control group, model group, and treatment group were killed, and the peripheral blood was collected. CTLs were sorted by CD3^+^CD8^+^ cell labelling in the peripheral blood. The selected CTLs were analyzed, revealing that perforin, granule B, and PD-1 mRNA and protein expression significantly increased. Perforin, granule B, and PD-1 expression was downregulated in the treatment group, indicating that the Chinese medicine improved ALF symptoms by inhibiting CTL function (Figures 2 and 3). In addition, these results suggested that Jiedu Huayu granules affected the killing efficacy of CTLs, thus alleviating CTL-mediated immune damage.

### 3.3. Inhibition of Hepatocyte Apoptosis and Liver Immune Response

**Figure 4:**
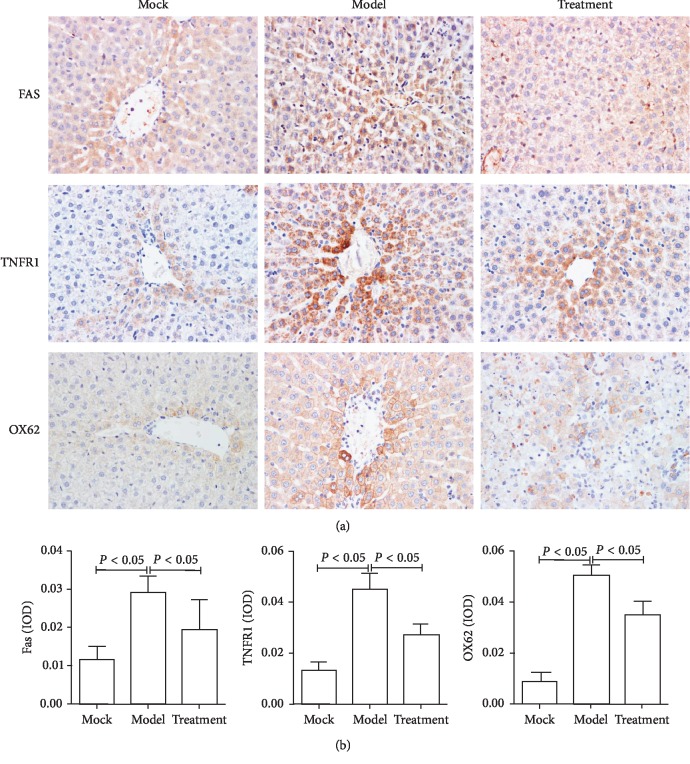
Fas, TNFR1, and OX62 protein expression in rat liver cells detected by immunohistochemistry. (a) Rats were killed, the liver was collected, and Fas, TNFR1, and OX62 were detected by immunohistochemistry (400x magnification). (b) IPP6.0 software was used to analyze the optical density of the staining in the immunohistochemical images. Three 400x magnification images were selected from each section for optical density analysis.

Next, to detect apoptosis and immune response in liver tissue, three rats from the control group, model group, and experimental group were randomly selected and sacrificed. The liver tissue was collected and sliced, and immunohistochemical analysis to detect hepatocyte apoptosis related proteins OX62, FAS, and TNFR1 was performed. The results showed that these proteins were highly expressed in the model group. The signal intensity in the treatment group was similar to that in the control group, suggesting that the treatment with Jiedu Huayu granules reduced liver cell apoptosis (Figure 4). These experimental results are also described in our previous article [18]. It is well known that CTL cells can induce apoptosis through FAS and TNFR1, while OX62 is closely related to maturity of CTL cells. Thus, these results suggested that Jiedu Huayu granules reduced the expression of OX62, therefore affecting the maturation of CTL. In addition, it reduced the expression of FAS and TNFR1 to inhibit hepatocyte death, which is associated with the mechanism of Jiedu Huayu granules inhibiting CTL function.

## 4. Discussion

Immune damage can be triggered by multiple factors and is the main cause of liver failure. The immune system protects against the invasion of various pathogens and maintains the stability of the internal environment. However, under pathological conditions, excessive activation or incorrect activation of the immune system can be deleterious to the body [19]. The liver is not only the biochemical plant of the body but also an endocrine organ. Some scientists also regard the liver as an immune organ; thus, the liver is often the first to bear the brunt when the immune system damages the body. The chemical damage and immune damage of hepatocytes often occur at the same time. They complement each other under physiological conditions, protecting the body against the invasion of pathogenic microorganisms and other damages. However, under pathological conditions, they cause a vicious circle and aggravate liver cell damage [20].

In this work, the role of Jiedu Huayu granules in the immune system against liver injury was explored. As a traditional Chinese medicine, it is interpreted as detoxification and used in severe cases of heat and jaundice, severe hepatitis, hyperbilirubinemia, and other diseases [21]. However, the interpretation of Chinese medicine regarding the effect of Jiedu Huayu granules is not giving any information regarding the mechanism of action ascribed in Western medicine. At present, more and more studies proved the mechanism of action of the drug to treat liver damage such as antioxidant activity, inflammation reduction, and apoptosis inhibition. It has also been shown to protect the liver in clinical trials, and it is widely used against liver fibrosis [22]. Our research revealed that the Jiedu Huayu granules are especially acting in the immune system.

With the continuous improvement of the basic and clinical research in liver diseases, the understanding of the etiology of diseases, causes, and treatments have also made great progresses, and the clinical treatment on liver diseases has been widely recognized and promoted. For example, the treatment against viral hepatitis changed from a simple treatment of liver-protecting enzymes to the current antiviral treatment and has achieved good clinical results. With the continuous development and promotion of antiviral treatments, some people believe that an antivirus is enough to resolve a viral disease [23]. To solve all the problems of hepatitis B and C, liver protection treatment seems to be a bit redundant [24, 25]. However, the understanding of the pathogenesis of liver diseases revealed that the virus itself does not cause liver damage but is the immune system to be involved. Therefore, the use of antivirus and hepatoprotective agents is more beneficial.

## Data Availability

The data used to support the findings of this study cannot be shared at this time as the data also form part of an ongoing study.
